# Supplementary material for: Obese Asthma Phenotype Is Associated with hsa-miR-26a-1-3p and hsa-miR-376a-3p Modulating the IGF Axis
Source: Int J Mol Sci. 2023 Jul 18;24(14):11620. doi: 10.3390/ijms241411620 (PMC10380435; doi:10.3390/ijms241411620)
Supplement: Supplementary file 1 [file ijms-24-11620-s001.zip › Gil-Martínez-miRNAs and IFG axis-Tables S1a y S1b IJMS modified version.pdf]

Table S1a. Demographic, inflammatory, and clinical characteristics of the study subjects, asthmatic and control individuals (population 1; lung biopsy samples analysis).

	**Asthmatics (n=16)**	**Controls (n=20)**	**P-value**
**Age (years)^**	52.4 (± 17.7)	63.7 (± 8.5)	*
**Female (%)**	6 (37.5%)	8 (40%)	N.S.
**Smoking habit (%)**			
**Smokers**	4/15 (26.7%)	4 (20%)	N.S.
**Ex-smokers**	7/15 (46.7%)	4 (20%)	N.S.
**Non-smokers**	4/15 (26.7%)	12 (40%)	N.S.
**Neutrophils (%)^**	71.4 % (± 16.2)	80.6 % (± 6.1)	N.S.
**Lymphocytes (%)^**	18.2 % (± 12.7)	10.7 % (± 3.9)	N.S.
**Monocytes (%)^**	8.4 % (± 2.9)	7.7 % (± 2.6)	N.S.
**Eosinophils (%)^**	1.6 % (± 1.9)	0.8 % (± 0.8)	N.S.
**Basophils (%)†**	0.3 % (0.1-0.5)	0.2 % (0.1-0.4)	N.S.
**Atopy (%)**	3 (18.8%)	0/18 (0%)	N.S.

Results are expressed as ^Mean (± SD) or †median (IQR); N.S., Non-significant; *, $p<0.05$.

Table S1b. Demographic, inflammatory, functional, and clinical characteristics of obese and non-obese asthmatic patients (population 2; serum samples analysis).

	Obese (n=26)	Non-obese (n=51)	P-value
**Age (years)^**	52.5 (±10.7)	46.7 (±15.5)	N.S.
**Female (%)**	19 (73.1%)	35 (68.6%)	N.S.
**BMI†**	33 (31.6-36.4)	23.8 (22.2-26.5)	****
**Obesity (%)**	26 (100%)	0 (0%)	****
**Tobacco habit (%)**			
**Smokers**	0/25 (0%)	3/47 (6.4%)	N.S.
**Passive**	2/25 (8%)	4/47 (8.5%)	N.S.
**Ex-smokers**	10/25 (40%)	15/47 (31.9%)	N.S.
**Non-smokers**	13/25 (52%)	25/47 (53.2%)	N.S.
**Blood eosinophils (cells/µL)†**	200 (115-525)	500 (100-700)	N.S.
**Sputum eosinophils (%)†**	4% (0-12)	2% (0-11.8)	N.S.
**Atopy (%)**	20 (76.9%)	33 (64.7%)	N.S.
**IgE (IU)†**	180.5 (65.3-460.8)	178 (62.9-401.5)	N.S.
**FEV_1/FVC (%)^**	86.5% (±14.6)	78.8% (±13.5)	*
**FeNO (ppb)†**	24 (14.5-36.5)	33.5 (15.8-68)	N.S.
**Exacerbations (%)**	13 (50%)	26 (51%)	N.S.
**Severity (%)**			
**Severe**	8/25 (32%)	25/44 (56.8%)	*
**Moderate**	11/25 (44%)	9/44 (20.5%)	*
**Mild**	6/25 (24%)	9/44 (20.5%)	N.S.
**Intermittent**	0/25 (0%)	1/44 (2.3%)	N.S.
**ACT†**	21 (16.5-22)	21 (17-24)	N.S.
**ICS and LABA (%)**	25 (96.2%)	44 (86.3%)	N.S.
**Late asthma onset**	17/24 (70.8%)	34/43 (79.1%)	N.S.

Results are expressed as ^mean (±SD) or †median (IQR); N.S., Non-significant; ****, $p<0.0001$; *, $p<0.05$; BMI, Body Mass Index; FEV_1, Forced Expiratory Volume measured during the first second; FVC, Forced Vital Capacity; FeNO, Fractional exhaled Nitric Oxide; Ppb, parts per billion; ICS and LABA, Inhaled Corticosteroids and Long-Acting β2-Agonists; ACT, Asthma Control Test.
