# Supplementary figures and images for: Obese Asthma Phenotype Is Associated with hsa-miR-26a-1-3p and hsa-miR-376a-3p Modulating the IGF Axis
Source: Int J Mol Sci. 2023 Jul 18;24(14):11620. doi: 10.3390/ijms241411620 (PMC10380435; doi:10.3390/ijms241411620)

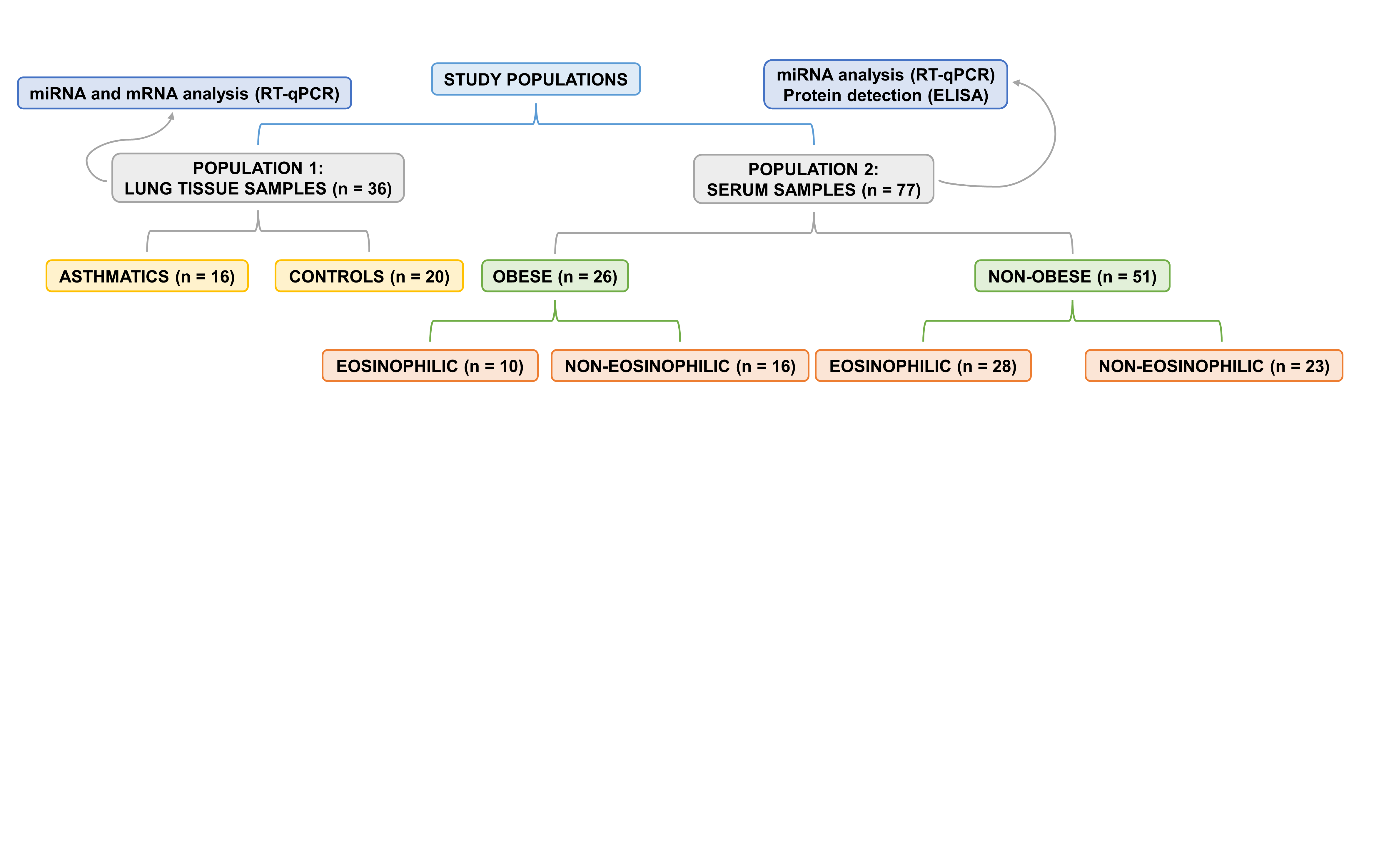

Supplement: Supplementary file 1 [file ijms-24-11620-s001.zip › Gil-Martínez-miRNAs and IFG axis-Figure S1.tif]
